# Subjective decision threshold for accurate visual detection performance in rats

**DOI:** 10.1038/s41598-018-27696-4

**Published:** 2018-06-19

**Authors:** Yuma Osako, Yoshio Sakurai, Junya Hirokawa

**Affiliations:** Laboratory of Neural Information, Graduate School of Brain Science, Doshisha University, 1-3 Tatara Miyakodani, Kyotanabe, Kyoto 610-0394 Japan

## Abstract

The dissociation between a subjective-criterion performance and forced performance in a sensory detection can provide critical insights into the neural correlates of sensory awareness. Here, we established a behavioral task for rats to test their spatial-visual cue detection ability, using a two alternative choice task with and without a third choice option where animals get rewards only in the objective absence of a visual cue. In the trials without the third option, spatial choice accuracy decreased from near perfect to near chance levels as the visual cue brightness decreased. In contrast, with the third option, the rats exhibited >90% spatial choice accuracy regardless of the cue brightness. The rats chose the third choice option less frequently when the cue was brighter, suggesting that rats have a generalized strategy to make spatial choices only when their internal detection criterion is met. Interestingly, even when the animals chose the third option, they could still significantly and correctly choose the direction of the visual stimuli if they were forced. Our data suggest that the rats’ variable detection performance with identical set of stimuli is derived from stochastic processing of visual signals with a certain internal detection threshold rather than general motivational threshold.

## Introduction

Subjective perception is often contrasted with objective perceptual performance^1–3^, suggesting that perceptual awareness is not always an accurate monitoring process of one’s own capability. A striking example of this distinction is patients with damage to their primary visual cortex (V1) who have the capacity to detect or discriminate visual stimuli without conscious perception^4–6^. This phenomenon is called “blindsight,” and the dissociation between conscious perception and behavioral performance suggests that humans process visual information without subjective visual awareness. Macaque monkeys with unilateral V1 lesions perform similarly to human blindsight subjects in various behavioral tasks^7–14^, suggesting the generality of the phenomenon across species. Clarifying the neural correlates of dissociation between subjective visibility and objective behavioral performance is key to understanding the neural mechanisms of generating metacognition and consciousness^15–19^. To this end, establishing a rodent model for quantitative evaluation of dissociation between subjective and forced performances would be useful for extending research to the molecular and neural circuit levels.

Recent behavioral studies have provided evidence for metacognition in rats^20–22^. Foote and Crystal set up two types of trials in which rats could decline the test (choice trial) or were forced to take the test (forced trial) in a sound duration–discrimination task. Because rats in the choice trials could only take the test when they were confident of a correct choice, the accuracy in these trials was greater than in the forced trials. Indeed, the proportion of rats that declined the test in the choice trials increased with increasing difficulty of discrimination. They concluded that rats had the capacity to use their internal state for behavioral performance, which could be called metacognition. Kepecs *et al*. established a theoretical framework for estimating the degree of metacognition (i.e., decision confidence), and proposed a trial-by-trial instantaneous measurement of decision confidence in an odor mixture categorization task in rats^23–26^. These studies demonstrated the strength of quantitative measurement of metacognition for clarifying the neural correlates of such internal variables^23,27^.

Although post-decision wagering – such as the waiting time paradigm – is useful for measuring metacognition as a quantitative variable^28,29^, awareness itself can be a binary phenomenon^30,31^. The subjective presence of perception, i.e., awareness, is dynamically influenced in an all-or-nothing manner by experimental manipulations such as top-down attention^32–36^, multisensory integration^37–39^ and inter-hemispheric interactions^40–42^ despite the continuous physical presence of stimuli. This implies separable neural substrates of awareness and decision confidence. However, in rodents, it is not known whether they have an internal detection criterion dissociated from forced performance.

In our previous studies using rats, we showed that visual detection performance is modulated by the animals’ internal states, such as top-down attention and multisensory integration^43–46^. These studies raised the question of whether rats could reliably utilize internal visual information. To clarify whether rats have internal criterion-based visual detections dissociated from criterion-independent visual detections, we compared the detectability of identical sets of spatial visual stimuli with and without a third choice option, which affords animals the opportunity to report the subjective absence of a visual cue. Note that animals were rewarded by choosing the third choice option only when the visual cue was not actually emitted. Therefore, optimal strategy in this task is to make the peripheral choice as long as the rats detected a peripheral visual cue regardless of the availability of the third choice option.

## Results

### Comparison between 3C and FC trials

**Figure 1:**
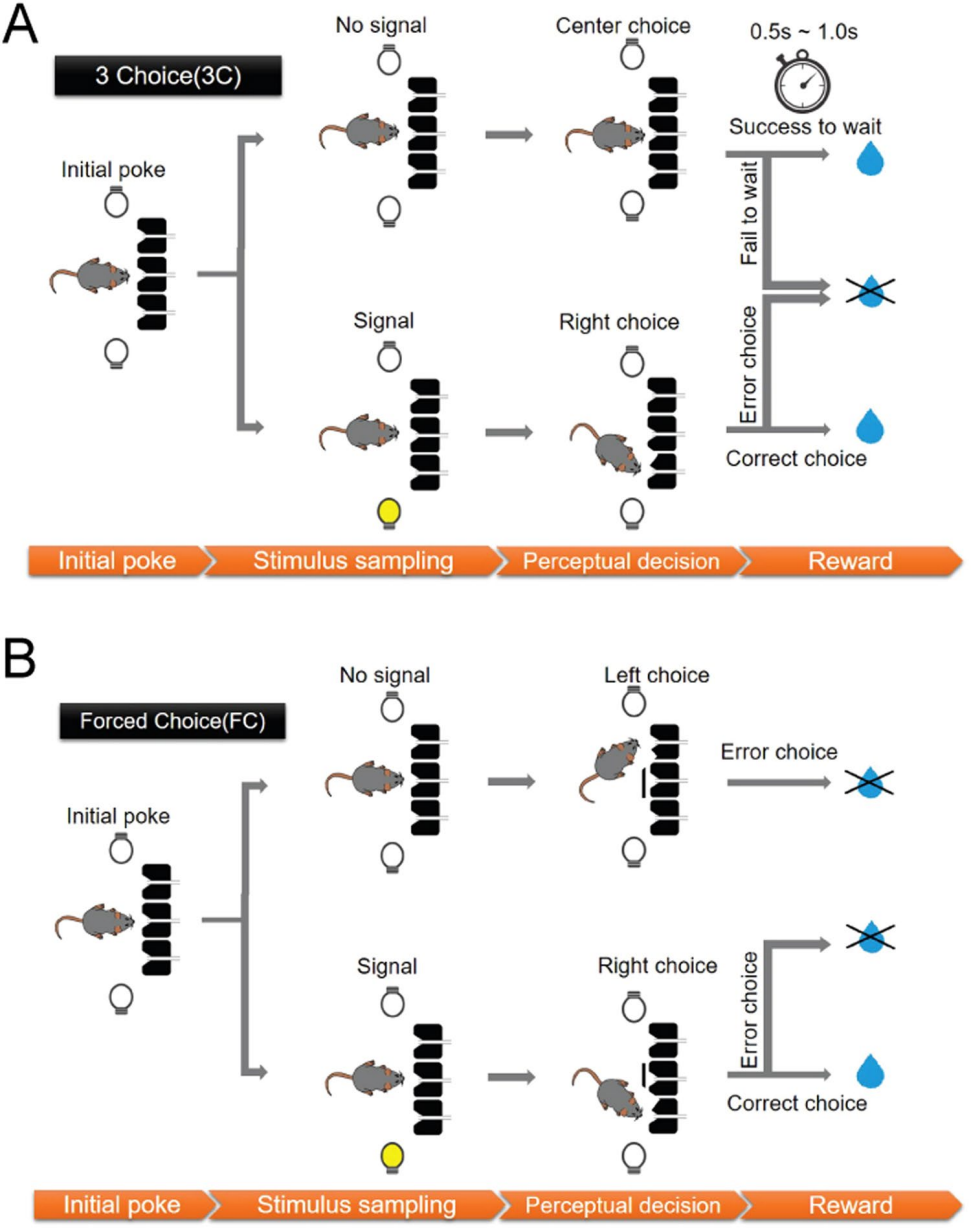
Schematics of sequences of behavioral and sensory events in the visual detection task. (**A**) Schematic of 3C trials. To start a trial, the rat had to poke its nose into the central port and, after a pseudorandom delay of 0.2–0.6 s, the visual stimuli was presented on the left or right LED for 0.2 s (Signal) or not presented (No signal). If rats chose the same direction as the stimulus, a water reward was given immediately in the chosen port. When no visual stimuli were presented, the reward was given after a 0.5–1.0 s nose poke held in the central port. (**B**) Schematic of FC trials. The sequence and condition of stimulus presentation were identical with 3C trials except that the central port becomes not available by closing shutter 0.5 s after stimulus presentation.

The rats initiated each trial by making nose pokes into the central port and the visual stimulus was presented (or not presented) from the left or right side after a 0.2–0.6 s random delay (Fig. 1A). The task was comprised of randomly interleaved three choice (3C) and forced-choice (FC) trials with equal probabilities in a session (see Methods in details). The only difference between the trial types was that, in FC trials, the central port was shut 0.5 s after the stimulus presentation timing with the shutter door to prevent the rat from continuing to central nose poke (Fig. 1).

**Figure 2:**
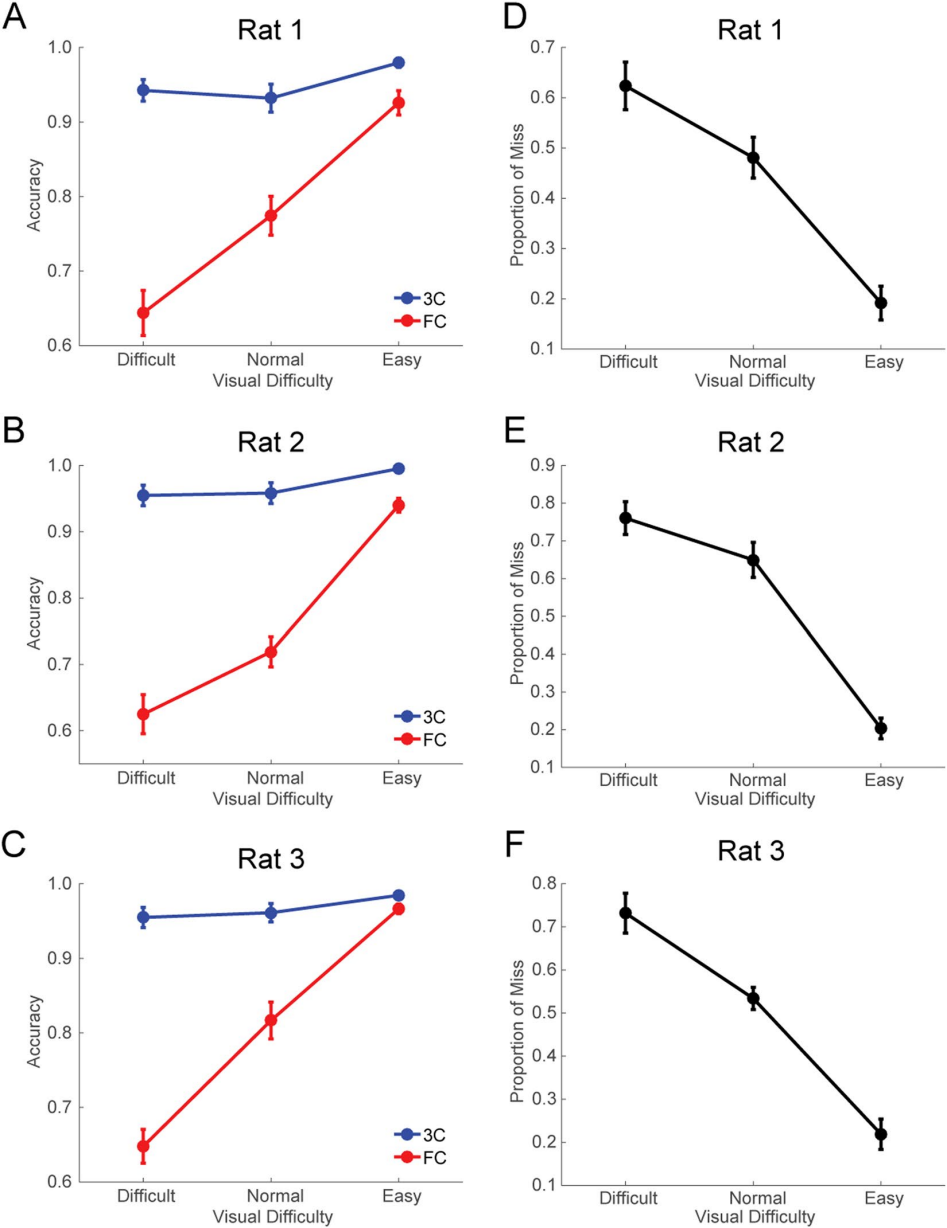
Behavioral performance in the visual detection task. (**A**–**C**) Choice accuracy is shown as mean ± SEM. (**D**–**F**) Proportion of misses is shown as mean ± SEM.

The choice accuracy in FC trials decreased from approximately 90% to 65% as the visual brightness decreased (Fig. 2A–C). By contrast, the choice accuracy was maintained at >90% regardless of the decrease in visual brightness in the 3C trials (Fig. 2A–C), while the rats missed the visual stimuli more often (from 20% to 80%) as the visual brightness decreased (Fig. 2D–F). The false alarm rate in the 3C trials was less than 5% on average (mean ± SEM: 3.69% ± 0.89 (n = 3)) when the visual stimulus was not presented. These results indicate that the hit rate in 3C trials reflects the probability that the rats recognized the visual cue.

### Reaction time between 3C and FC trials

**Figure 3:**
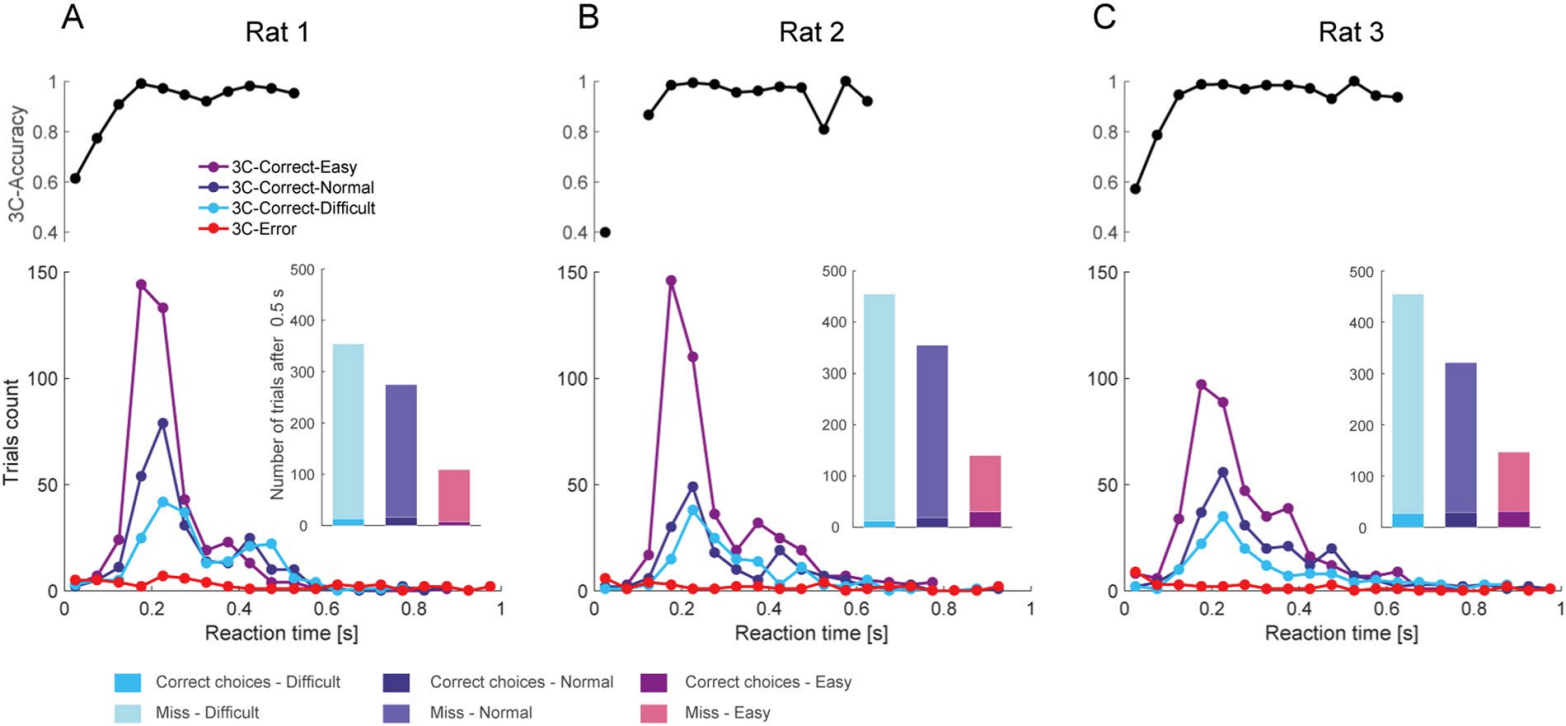
Reaction time distribution and detection accuracy in the 3C trials. (**A**–**C**) The distributions of reaction times of the spatial choices for correct in three visual difficulty (Easy: purple, Normal: dark blue, Difficult: light blue) and error (red) trials for each subject (bottom). The inset shows the number of correct choices and miss trials with reaction time more than 0.5 s. Spatial choice accuracy for each reaction time bins is shown above (with minimum trial number of 10 trials).

**Figure 4:**
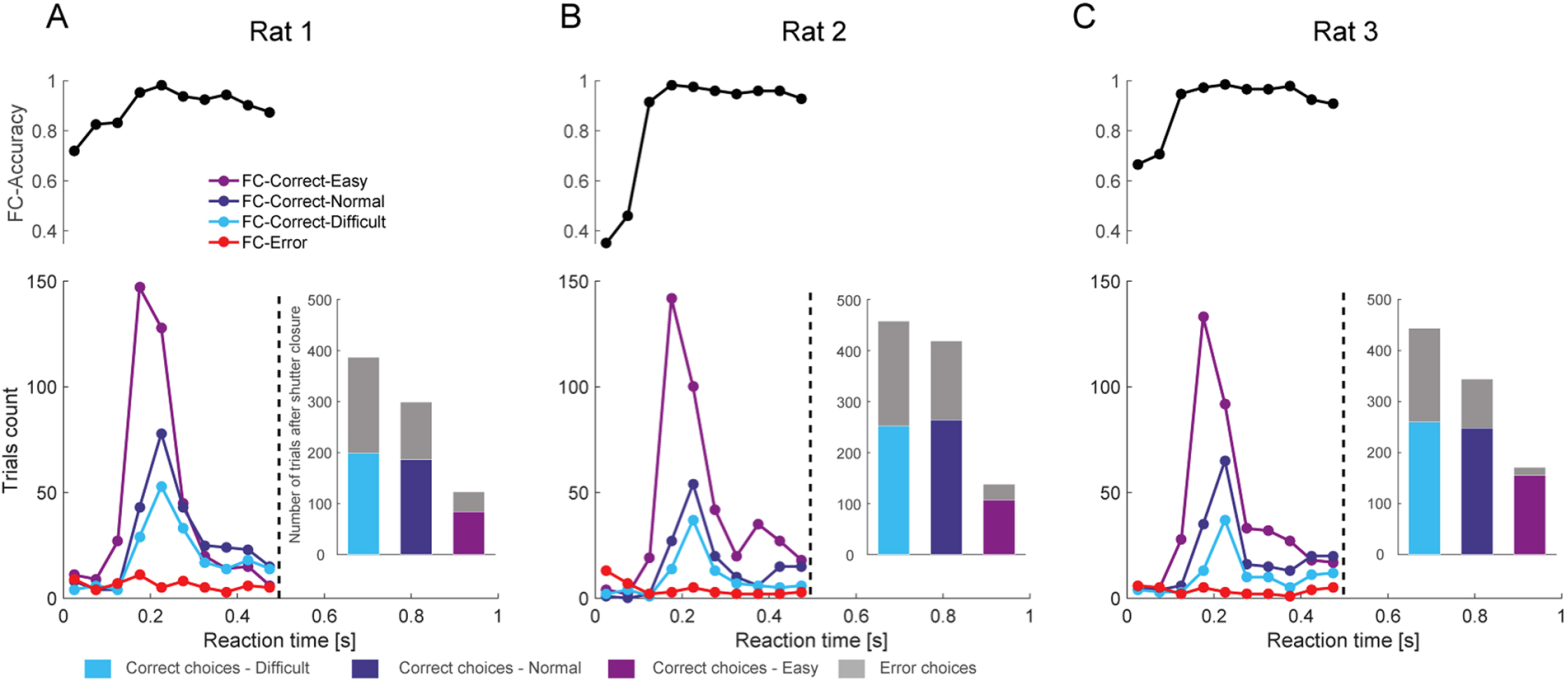
Reaction time distribution and detection accuracy in the FC trials. (**A**–**C**) The distributions of reaction times of the spatial choices for the correct in three visual difficulty (Easy: purple, Normal: dark blue, Difficult: light blue) and error (red) trials for each subject (bottom). The inset shows the number of correct and error choices after the shutter closure (0.5 s). Spatial choice accuracy for each reaction time bins is shown above (with minimum trial number of 10 trials). Note that only the reaction time before shutter release (<0.5 s) is shown.

To understand detection performance relative to the shutter closure timing, the reaction times of the behavioral responses in the 3C and FC trials were analyzed along with detection accuracy. In 3C trials, the reaction times for correct choices have a sharp peak at around 0.2 s (Fig. 3), showing that highly stereotypic responses to visual stimuli are repeated when the rats make correct choices. The reaction time distribution was slightly shifted to earlier time points in easier trials. By contrast, reaction times in erroneous trials were scattered without a specific peak, suggesting that incorrect choices were made at random without a specific clue. The choice accuracy in the 3C trials reached 100% at around 0.2 s and was maintained near 100% by at least 0.5 s (Fig. 3. Upper, rat1 and rat2), whereas a prolonged tail of reaction time distribution (up to 1 s) was observed in rat3. Overall, more than 95% of correct-responses were made before 0.5 s in 3C (rat1: 97.89%, rat2: 97.12%, rat3: 96.25%, Table 1 in detail). As expected, there were no significant differences between the 3C and FC trials before the expected time for the shutter closure for each stimulus condition (*p* > 0.05, Kolmogorov-Smirnov test, Fig. 3 and Fig. 4). The reaction time analysis shows that the degraded accuracy in FC trials is mostly derived from the forced choices after the shutter closure.

**Table 1:** Number of peripheral choices in 3C and FC trials.

Rat Number	Trial Type	Number of peripheral choices **less** than 0.5 sec	Number of peripheral choices **larger** than 0.5 sec
Rat 1	3C	881	52
	FC	944	809
Rat 2	3C	707	72
	FC	696	1015
Rat 3	3C	749	92
	FC	730	959

### Detection accuracy after the shutter closure

**Figure 5:**
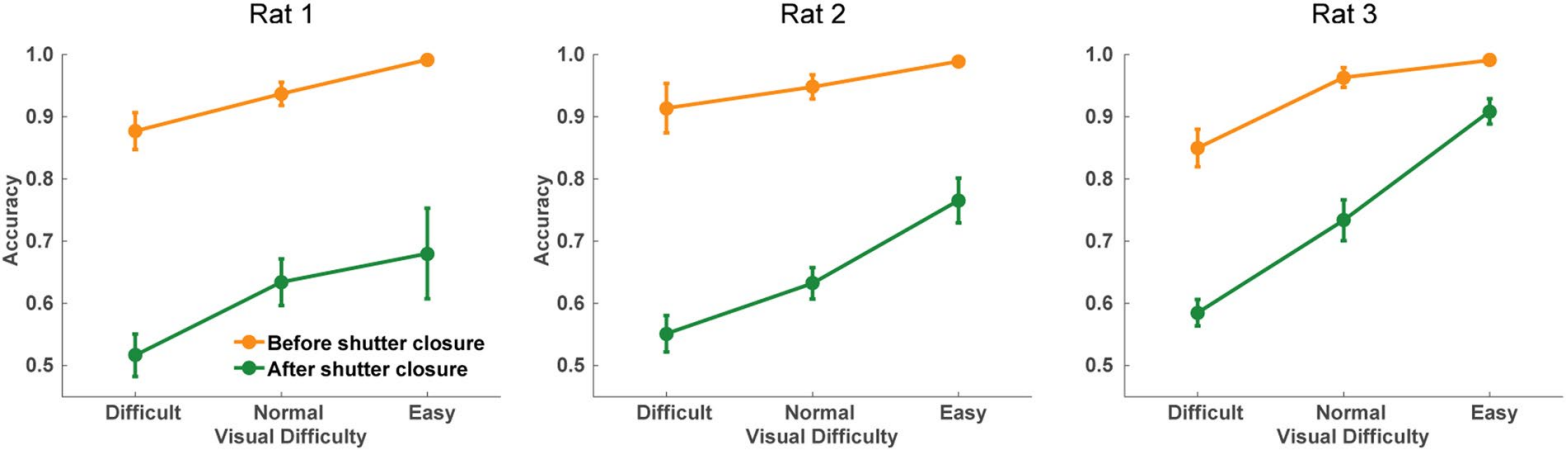
Choice accuracy of trials after the shutter closure. Choice accuracy before (green) and after (yellow) the shutter is closed as expressed as mean ± SEM.

Next, we examined whether the forced choice after the shutter closure was by chance (50%) or above chance. Surprisingly, the rats still significantly chose the correct side in a visual difficulty-dependent manner; All three rats significantly chose the correct side in easy and normal conditions (Fig. 5, t-test from chance level); one animal (rat 3) performed above chance level in the difficult condition, while the others (rat 1 and rat 2) performed at the chance level in the difficult condition (Fig. 5). In addition, the proportion of correct choices after 0.5 s of the stimulus onset in FC was much larger than those in 3C (insets in Figs 3 and 4), suggesting that the residual accuracy after the shutter closure in FC is not explained by the correct choices with slow reaction time observed in 3C. These results suggest that the animals sometimes failed to make hit-responses even though they could choose the correct side if forced. We note that there is a weak trend of the decrease in choice accuracy before the shutter closure in FC in difficult trials (compare accuracies before the shutter closure in Fig. 5 and those in 3C in Fig. 2). However, the direct comparison between the accuracies between 3C and FC before the shutter closure were not significant (*p* > 0.05, one-way ANOVA and Tukey’s test for post-hoc comparison), except for one animal (rat1: *p* = 0.03 in difficult trials), suggesting the effect is due to subtle experimental fluctuation (see Discussion). We then asked if the failure of the hit-response before the shutter closure in eventually-correct trials was due to general procrastination (including longer reaction time) or degraded visibility. If the former is the case, failed hit-responses should occur independently of the visual difficulty, and therefore the ratio of correct responses after the shutter closure relative to all correct responses should be constant across visual difficulty levels. Our data did not support this possibility. Instead, failed hit-responses significantly decreased with increasing visual difficulty (analyzed in a one-way ANOVA for each visual difficulty level and a post-hoc test adjusted for multiple comparisons), supporting the notion that animals stayed in the central port by the time the shutter closes due to degraded visibility.

## Discussion

In this study, we developed a behavioral paradigm for rats to detect spatial visual cues with graded brightness and then examined how their detection performance with and without a third choice option were dissociated. Our results demonstrated that rats have a generalized criterion in reliably detecting visual stimuli with different luminance and that it cannot be explained by general motivational threshold, arguing for the presence of visual awareness in rats. The results are generally consistent with previous findings in the sense that rats can utilize their internal state for subsequent decision-making^20,22,23^. Our task was also designed to be compatible with multiunit recordings and optogenetics with a complete experiment for each session with multiple randomly-interleaved conditions, which is potentially useful in the search for neural correlates of visual awareness.

Previous studies have demonstrated that rats have the ability to utilize decision confidence in sound^20^ and odor categorization^23,25^ tasks. However, a perceptual categorization task does not guarantee categorically distinct awareness because the decision boundary for the categorization is arbitrarily set by the experimenters regardless of the rats’ subjective perception. In addition, uncertain responses owing to “intermediate” stimuli are often confounded by reinforcement or economic strategies without requiring metacognition^47–50^. In this study, we utilized a binary visual detection task where such “intermediate” cues were not available: rats were rewarded in peripheral ports when visual stimuli were actually emitted from either the left or right side, or they were rewarded in the central port when visual stimuli were not emitted. Thus, there was no ambiguity in stimulus-reward association unlike in the sensory categorization task described above. Indeed, the spatial choice accuracy was consistently >90% regardless of the visual brightness in shorter reaction time trials (<0.5 s), whereas the overall accuracy decreased to 60% in low visual brightness in the FC trials (Figs 2, 3 and 4). This suggests that rats have a generalized strategy to choose the peripheral ports when their internal detection criterion is met.

**Figure 6:**
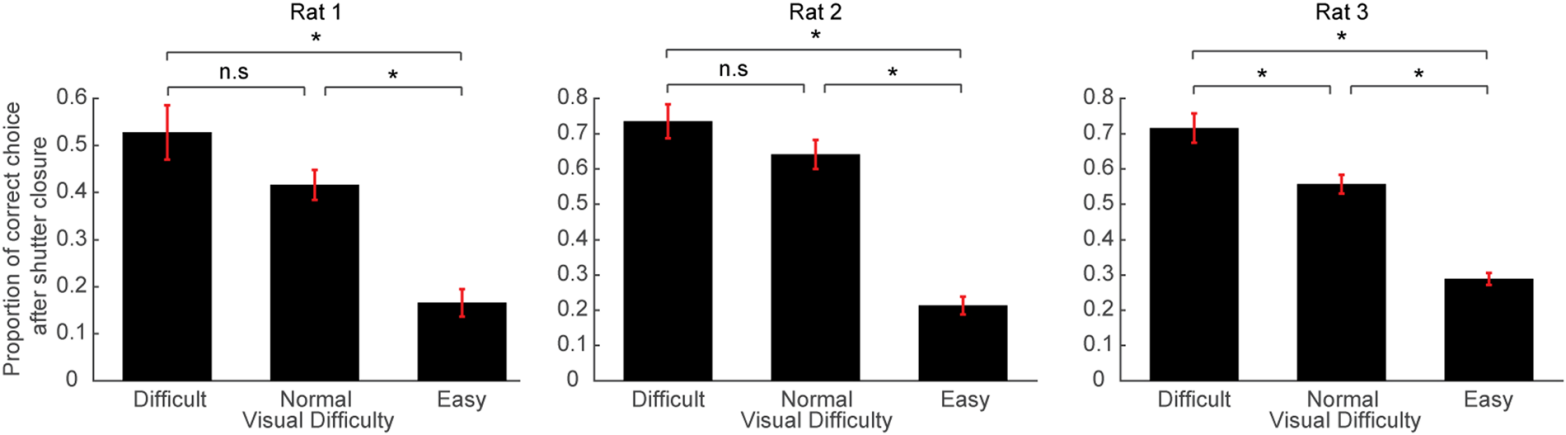
Proportion of the correct trials after the shutter closure in all the correct trials in FC trials. Proportion of correct trials after the shutter closure in all correct trials (before and after the shutter closure) is shown as mean ± SEM. For the analysis of data across conditions, one-way ANOVA and Tukey’s test for post-hoc comparison were used. *p < 0.05.

It has been shown that the difference in the performance between a yes-no detection task and a forced choice task disappears in human subjects when considering decision bias (with some exceptions such as the “blindsight” cases). Therefore, one may argue that the difference in accuracy between 3C and FC in our task may be also explained by decision bias rather than detectability difference. Consistent with this idea, our data suggest that the difference is mainly from conservative decision criterion in 3C. Because the 3C and FC are essentially indistinguishable for animals until the shutter closure as designed, the discriminability and the bias should be also same between 3C and FC at least by the time of 0.5 s after the stimulus onset. Therefore, the high accuracy in 3C reflect the above-criterion choice whereas the degraded choice accuracy in FC is sub-threshold forced choice. Though we observed a weak decrease of choice accuracy in difficult trials before the shutter closure in FC (Fig. 5), the significant effect was only found in one animal, likely due to a subtle conditional fluctuation such as unreliable mechanical onset of the shutter closure. We think the effect (~5%) is negligible considering the large dissociation of the accuracy (~35%) observed in 3C and FC (Fig. 2). In addition, it is unlikely that the minor decrease of the accuracy contributes to the increased accuracy after the shutter closure (Fig. 6). Therefore, we conclude that the difference in spatial accuracy in FC and 3C is simply derived from the conservative detection criterion in 3C.

Though signal detection theory provides one of the solutions to disambiguate the effect of detectability and the response criterion in an objective way^51,52^, it is hard to apply signal detection theory directly to our dataset because of the complexity of our task design. One drawback to applying signal detection theory to the study of awareness in animals is that it requires the experimenters to obtain multiple data points with different decision criteria. As a result, the animals might adaptively adjust their decision criterion based on their motivational state and therefore their subjective report may no longer reflect their sensory awareness. In our study, we trained animals to minimize their false alarm rates (i.e., go response without signal) by reinforcing them to maintain a central poke as the default state. As a result, we obtained reliable go-responses with high discrimination accuracy (~95%, Fig. 2) regardless of the stimulus intensity and a low false alarm rate (<5%), demonstrating that rats are capable of a highly reliable subjective report of their visual detection across different signal levels.

While animals could choose the correct side with near perfect accuracy (>90%) when they made a hit-response, they missed the same stimuli so often, especially for low brightness (60–80%). What causes this response variability? In our task setting, the animals maintained their heads in the central port while the visual stimuli were delivered from identical peripheral positions. Therefore, one possibility is that the missed trials may be due to decreased visual signals given a conservative threshold due to the animals’ fluctuating internal states (e.g., visibility, confidence) and/or physical changes in light reception on their retina. The alternative possibility is that it is due to a general conservativeness or bias to the central port unrelated to signal levels. To discriminate between these possibilities, we introduced trials where the animals were forced to choose the direction of the cue (i.e., FC trials) and analyzed a fraction of the choices where animals failed to make hit-responses by the time of the shutter closure. To our surprise, the rats could still make correct choices depending on the strength of the visual stimuli (Fig. 5). Importantly, the occurrence of correct choices after the shutter closure was not explained by randomly omitted/procrastinated hit-responses (i.e., center bias), but rather was anti-correlated with stimulus intensity (Fig. 6). The results indicate that the variable detection performance to identical stimuli is derived from stochastic internal processing of visual signals with a certain internal threshold.

Whether or not such an internal threshold is related to the decision itself (e.g., payoff^51,53^, confidence^23^, expectation^13^) or to subjective visibility is not directly demonstrated in our experiments. For instance, it has been shown that frequent reward condition lowers the decision criterion of subjects, whereas difficult task increases the decision criterion^54^. Thus, animals may adjust their decision criterion based on overall task difficulty rather than faithfully following their vision. Note that such internal threshold is different from the fluctuation of general motivation discussed (Fig. 6) because it depends on sensory evidence. In addition, it is also possible that the optimal threshold was adjusted by the tradeoff between reward values and the reward likelihood estimated trial-by-trial from internal visual signals regardless of the visual awareness. It is possible to differentiate these possibilities by systematically changing the center reward value and seeing how the spatial choice accuracy and false alarm rates for 3C would change. However, we emphasize that considering the fact that the rats were never rewarded by remaining in the central port in the presence of stimuli and there is no objective ambiguity in the stimulus-reward association, there is little room for trading between detection confidence and the center reward value. Therefore, it is more plausible that the threshold is related to visibility rather than an adaptive decision criterion.

Compared with the dissociation reported by Foote & Crystals (2007), the extent of dissociation in our study was larger and more robust (compare Fig. 2E,F, and G in Foote & Crystals (2007) and Fig. 2 in the current study). The reason for this discrepancy can be largely explained by differences between awareness and confidence reports. Confidence reports for categorization rely on the degree of difference in the competing evidence, and the forced categorization may use the sign of the evidence. Therefore, confidence reports and forced performance are unavoidably correlated with each other. In contrast, awareness reports rely on the threshold of sensory evidence; subjects will report otherwise as long as the sensory evidence does not reach the threshold. Once the sensory evidence reaches the threshold, the report is reliable since it is supported by a high degree of sensory evidence. Therefore, the dissociation will be large in the perceptual awareness report task.

It is intriguing to consider what neural mechanisms enable above-threshold vision to be dissociated from sub-threshold vision. It is advantageous to utilize rodents as model animals given the number of molecular biology tools available. We also note that our behavioral setup utilizes a commercial standard operant box with nose pokes and an open-source behavioral control system (see Methods for details), which is readily available to the community. On the other hand, a disadvantage of the current protocol is the lack of precise control of the visual stimuli on retina, which is a standard procedure in non-human primate studies. Random presentations of visual stimuli on the retina would inevitably cause random noise in the behavioral results, causing difficulty in the interpretation of neural correlates of subjectivity. One way to overcome this issue is to utilize head-fixed animals^55^, head-mounted goggles^56^ or direct activation of sensory neurons^57^. Future studies dealing with precise control of stimulation would be useful for studying neural correlates of visual awareness in rodents.

## Materials and Methods

### Animals

Five male Long–Evans rats (Shimizu Laboratory Supplies, Kyoto, Japan) aged 12–17 weeks and weighing 250–500 g at the beginning of the training were individually housed under standard laboratory conditions in a light and dark cycle (lights on at 8:00 and off at 21:00) with food freely available. The rats were placed on a liquid restriction schedule with daily body weight monitoring to ensure that body mass remained within 85% of prior mass before restriction. The rats received water during each behavioral session and ad libitum in the 10 min after the session in their home cage. Two out of five rats were excluded from analysis because they could not achieve the training criterion in 3-choice training phase within our limited time. All experiments were performed in accordance with the guidelines for animal experiments at Doshisha University with the approval of the Animal Research Committee of Doshisha University.

### Apparatus

The behavioral apparatus was as previously described^46^ with modifications for the water reward^58^ using Bpod and PulsePal (Sanworks LLC, NY, USA), which are open source TTL event measurement and control devices designed for behavioral tasks^59^. Our system comprises identical operant chambers (O’Hara & Co., Tokyo, Japan), each located in a soundproof box (Brain Science Idea Co. Ltd., Osaka, Japan), with three nose poke ports in the front wall and a shutter door for the central port. The three ports were equipped with interior illumination (white light-emitting diode (LED)) and infrared photodiodes, and interruption of the infrared beam signaled port entry with a TTL pulse. A water reward could be delivered from the gravity-fed reservoirs, which were regulated by solenoid valves (The Lee Company, CT, USA). The reward amount, which was determined by the solenoid valve opening duration, was set to 0.01 ml and regularly calibrated. The shutter door for the central port was controlled by Arduino Due (Italy). Visual stimuli were presented on either the left or right side where the rats’ eyes were directed when they poked the central port^46^. The visual stimulus was a white LED (4000 mcd; RS components, Japan) covered by a frosted plastic diffuser to generate homogenous illumination.

### Visual cue detection task

The task design was based on our previous study^46^, with modification of the free choice and FC paradigms from Foote & Crystal (2007). The task was comprised of randomly interleaved three choice (3C) and forced-choice (FC) trials with equal probabilities in a session. The only difference between the trial types was that, in FC trials, the central port was shut with the shutter door to prevent the rat from continuing to central nose poke (Fig. 1). After a fixed 2.5 s inter-trial interval (ITI), the central port was illuminated by an interior LED of the central port signaling the ready state of a trial initiation. The rats initiated each trial by making nose pokes into the central port. After a 0.2–0.6 s random stimulus delay, the visual stimulus was presented from the left or right side for a duration of 0.2 s. Rats were allowed to make a choice response after the end of the stimulus delay period. The trials where animals prematurely left the port before stimulus delay were canceled and they needed to re-initiate the trials. We randomly provided one of three levels of visual brightness (difficult, normal, and easy) for each trial by modulating the voltage ranging 0.02–5.1 lx. Difficult, normal and easy stimuli were selected for each subject such that the subject detected the stimuli with respective accuracy of 55%–65%, 65–80% and >80%, respectively, in the forced-choice (FC) trials. The probabilities for left, right, or no visual stimulus were equal (33% per condition) in 3C and FC trials (Fig. 1A). The reward was given if rats chose the same side where the visual stimuli was emitted in the 3C and FC trials. If animals kept nose poke more than X s in the central port after the presentation of the visual stimuli, the trial was treated as miss error. X were drawn from the uniform distribution with a range of [0.5, 1]. Note that X is aligned with the expected timing of the reward delivery in no-signal trials so that animals cannot utilize the absence of reward delivery as indication of the presence of unnoticed visual stimuli. Failure of the peripheral choices within 5 s after nose withdrawal from the central port was also treated as miss error, though it occurred rarely (<5%). There was no punishment in any error trials and next trial was allowed to be initiated after ITI. In the no-signal trials, animals need to wait for 0.2–0.6 s without stimulus and another 0.5–1 s to get reward from the central port. There was no cue to distinguish the initial delay (0.2–0.6 s) and reward delay (0.5–1 s). Thus, animals did not have any external clue to differentiate the signal trials from the no-signal trials, except for the presentation of the signal itself. In the FC trials, the shutter was closed 0.5 s after stimulus presentation onset, and the rats were forced to choose either the left or right port (Fig. 1B). In cases where no stimuli were presented in FC trials, the animals were never rewarded. After task training (see below in details) was complete, the visual detection task was tested for 10 sessions for each rat. Each session was terminated when the rats completed more than 500 trials, which usually takes 2 hours.

### Training procedure

#### Initial training

On the first day of training, rats were acclimated to the operant chamber. A water reward was given in the central port illuminated by an interior LED light. On the following day, the interior LEDs for the left and right ports were illuminated 0.1 s after the center poke in, and nose poke into those peripheral ports was immediately rewarded in the same port. The initial delay for illumination of the peripheral ports was extended from 0.2 to 0.6 s over the subsequent 2–4 days. The initial training phase conditioned the animals to poke the central port to initiate the trial (initial poke) and make a subsequent peripheral nose poke into the left or right port.

#### 3-choice training

In the next step, the peripheral visual stimulus was presented using either the left or right LED apparatus for 0.2 s. Animals were rewarded if they poked their nose into the peripheral port on the same side as the visual stimulus. Once choice accuracy exceeded the criterion (>90%), the interior LEDs were turned off except for the LED in the central port. In the next step of this training phase, the peripheral visual stimuli were not presented and the rats were trained to maintain a central nose poke for 0.5–1.0 s for 15 µl of water reward. In the last step, left, right, or no visual stimulus was presented with equal probability (33% per condition) in a pseudorandom order and animals were rewarded for making a left, right, or central choice, respectively. This procedure lasted 10–20 days to meet the criterion (Choice accuracy >90% and the central poking accuracy >90%).

#### Forced-choice training

In the next step of visual detection task training, FC trials were introduced. When the rats poked into the central port to initiate the trial, the shutter closed the central port 0.5 s after stimulus presentation. Once the shutter closed the central port, the rats were forced to nose poke either the left or right port since they could not maintain the central poke. No reward was given when the visual stimulus was not presented in FC trials. When the animals met the criterion of 80% choice accuracy, they advanced to the last stage of visual detection task training. This procedure lasted 5–10 days to meet the above criterion.

#### Mixed-choice training

In the final step of visual detection task training, both 3-choice and forced-choice trials were interleaved in a single session. Once animals achieved 90% accuracy in 3-choice trials and more than 80% accuracy in forced-choice trials, we introduced three grades of visual stimuli difficulty using different brightness levels. The brightness was modulated to be easy, normal, or difficult, as described above. This training lasted 10–20 days.

### Behavioral data analysis

All statistical analysis of behavioral data was conducted using MATLAB 2017a (Mathworks). Hit, miss, and false alarm rates were defined in 3C trials. The miss rate was the percentage of central port choices in trials where visual stimuli were presented; the hit rate was the percentage of peripheral port choices where visual stimuli were presented, regardless of the correctness of the choice side; and the false alarm was the percentage of peripheral port choices in trials where no visual stimuli were presented. Reaction time was defined as the duration from stimulus presentation onset to nose withdrawal from the central hole. Trials with a reaction time of less than 130 ms was considered as invalid, which correspond to 2.75 ± 0.8% of the total number of trials, were excluded from the calculation of the spatial choice accuracy as they were too early to have been responses to the stimulus^60^. For the analysis of data across the conditions, one-way ANOVA and Tukey’s test for post-hoc comparison were used. For analyzing the choice accuracy after shutter release in the FC trials (Fig. 5), the student’s t-test was used to compare chance levels (*p* = 0.5). All data are presented as mean ± SEM.
